# Exploiting Intersentence Information for Better Question-Driven Abstractive Summarization: Algorithm Development and Validation

**DOI:** 10.2196/38052

**Published:** 2022-08-15

**Authors:** Xin Wang, Jian Wang, Bo Xu, Hongfei Lin, Bo Zhang, Zhihao Yang

**Affiliations:** 1 School of Computer Science and Technology Dalian University of Technology Dalian China

**Keywords:** question-driven abstractive summarization, transformer, multi-head attention, pointer network, question answering, factual consistency, algorithm, validation, natural language processing

## Abstract

**Background:**

Question-driven summarization has become a practical and accurate approach to summarizing the source document. The generated summary should be concise and consistent with the concerned question, and thus, it could be regarded as the answer to the nonfactoid question. Existing methods do not fully exploit question information over documents and dependencies across sentences. Besides, most existing summarization evaluation tools like recall-oriented understudy for gisting evaluation (ROUGE) calculate N-gram overlaps between the generated summary and the reference summary while neglecting the factual consistency problem.

**Objective:**

This paper proposes a novel question-driven abstractive summarization model based on transformer, including a two-step attention mechanism and an overall integration mechanism, which can generate concise and consistent summaries for nonfactoid question answering.

**Methods:**

Specifically, the two-step attention mechanism is proposed to exploit the mutual information both of question to context and sentence over other sentences. We further introduced an overall integration mechanism and a novel pointer network for information integration. We conducted a question-answering task to evaluate the factual consistency between the generated summary and the reference summary.

**Results:**

The experimental results of question-driven summarization on the PubMedQA data set showed that our model achieved ROUGE-1, ROUGE-2, and ROUGE-L measures of 36.01, 15.59, and 30.22, respectively, which is superior to the state-of-the-art methods with a gain of 0.79 (absolute) in the ROUGE-2 score. The question-answering task demonstrates that the generated summaries of our model have better factual constancy. Our method achieved 94.2% accuracy and a 77.57% F1 score.

**Conclusions:**

Our proposed question-driven summarization model effectively exploits the mutual information among the question, document, and summary to generate concise and consistent summaries.

## Introduction

Automatic text summarization of natural language aims to summarize the source document to generate a concise and informative description for helping people efficiently and quickly capture the main idea [1,2]. In the biomedical domain, question-driven answer summarization can be particularly useful for people whether they have a biomedical background or not because the generated summary only covers the key information with respect to a specific question and filters out the explanation part [3]. It is different from a factoid question-answering (QA) [4] system. The answer of factoid QA is a phrase or a sentence according to the question, but users prefer the detailed answer including more information to the accurate answer. Summaries for nonfactoid questions [5] should be semantically consistent and identical with the context. PubMedQA [6] is a novel biomedical nonfactoid QA data set collected from PubMed articles in which the title is a question and can be answered by yes or no. Some related studies [7,8] treat this QA data set as a summarization task and take the conclusion part of the abstract as the answer summary.

Early works put emphasis on query-based summarization approaches [9-11] in which the aim is to extract the sentences relevant to the given query. However, these methods are typically based on semantic relevance from query to context and neglect mutual information at the sentence level, which is helpful for the reasoning or inference process in question-driven summarization. These traditional extractive summarization methods are mainly based on information retrieval methods to select sentences that heavily rely on feature engineering, and the results performance is restricted by pipelines [5,12,13]. Though extractive summarization is more grammatical and coherent, the extractive sentences fail to have a logical connection. In contrast to extractive methods, abstractive methods produce summaries at the word level based on semantic comprehension [8]. Consequently, question-driven abstractive answer summarization is studied to generate the concise and salient short answer, which is also informative for answering the question.

To tackle question-driven abstractive summarization, the answer summary should be highly related to the concerned question. Existing studies [7,8,14] often concentrate on processing the mutual information between the question and document. However, though some sentences are not strongly related to the question, they further explain the central entity in question and affect the expression of the context. Mutual information among answer sentences is underused. Furthermore, it is hard for the recurrent neural network (RNN)–based model to capture the information of long sentences. Existing studies model the sentences separately, which hinders the interaction among sentences. To this end, we propose a novel transformer-based model [15] named Trans-Att that incorporates a two-step attention mechanism to enhance the mutual information both of question to context and sentence over other sentences. A novel multi-view pointer-generator network is proposed to create a condensed and concise summary to better use the question and context information.

Furthermore, a common problem in the practical application of abstractive summarization models is the factual inconsistency [16]. This refers to the phenomenon that the model produces a summary that sometimes distorts and fabricates the facts. Recent studies point out that up to 30% of the generated summaries contain such factual inconsistencies [16,17]. One main reason is that most existing summarization evaluation tools calculate N-gram overlaps between the generated summary and the reference [16]. Though some models make higher scores in token-level metrics like recall-oriented understudy for gisting evaluation (ROUGE) [18], the generated summaries still lack factual correctness. Thus, human evaluation is still the primary method for evaluating the factual consistency. In question-driven answer summarization, generated summaries should be consistent with the context semantically. Wang et al [19] and Durmus et al [20] propose the QA-based factual consistency evaluation metrics QAGS and FEQA separately. They first generate a set of questions about the summary and then use a QA model to answer these questions for evaluation. Because of the characteristics of the PubMedQA data set, the questions are general questions, and they can be answered by yes or no. We use the summaries as the context for the QA task to evaluate the factual consistency.

In this paper, a novel question-driven abstractive summarization based on transformer is proposed, namely Trans-Att, that incorporates a two-step attention mechanism and an overall integration mechanism to summarize the document with respect to the nonfactoid questions. Concretely, the two-step attention mechanism can learn richer structural dependencies among sentences and the relevance of the question and the document. The overall integration mechanism integrates the question, the document, and the correlative summary to generate a summary representation, which allows the model to use the comprehensive information. A novel multi-view pointer network is then proposed by integrating transformer and pointer-generator networks [21] to facilitate copy words from the question or the document to better use the question and context information. Finally, besides question-driven abstractive summarization evaluated by ROUGE, we also assess the model performance by QA task to evaluate the generated summary and whether they are factually consistent with the source document with regard to the question. The effectiveness of this model is empirically validated on the text summarization task and QA task, and achieves state-of-the-art performance on the PubMedQA data set.

The following are our main contributions. First, the novel architecture Trans-Att uses a two-step attention mechanism for better integrating the information in both question to context and sentence over other sentences.

Second, we propose a novel multi-view pointer network to generate tokens through overall integration, which integrates the attentive question, the attentive document, and the correlative summary to generate a summary representation.

Finally, besides ROUGE for automatically evaluating the summarized answers, we conduct a QA task to evaluate the factual consistency.

## Methods

### Question-Driven Abstractive Summarization

Automatic text summarization is a challenging task in the natural language processing field. It aims to generate simple and coherent essays that comprehensively and accurately reflect the central content of an original document. It can be categorized into two approaches: extractive and abstractive methods. The former method selects a few relevant sentences from the original text, while the latter needs to rephrase and generate a new sentence in which some words are not necessarily present in the original text. In this paper, we focus on abstractive summarization for its potential of summarizing the text more coherently and logically.

Question-driven summarization is intended to summarize the original document in terms of a specific question, which is different from query-based summarization. In query-based summarization, the query is often a word or a phrase referring to a particular entity [11]. Whereas a question may contain several entities and a specific semantic meaning, and this requires the model to have the reasoning or inference ability to identify the corresponding semantic contents in question-driven summarization [8]. Early query-based summarization methods heavily rely on feature engineering including query-dependent features and query-independent features. The former includes named entity matching and semantic sentence matching, and the latter includes term frequency–inverse document frequency and stop word penalty [1,22]. Recently, some abstractive sequence-to-sequence neural networks have recently been proposed to generate summaries in regard to the given query [10,11]. Some recent works have developed a new method for question-driven summarization [7,8,14] in nonfactoid QA that requires much reasoning and an inference process. However, these methods only model the relation between the question and each sentence, and neglect the mutual information among sentences.

### Problem Formulation

**Figure 1:**
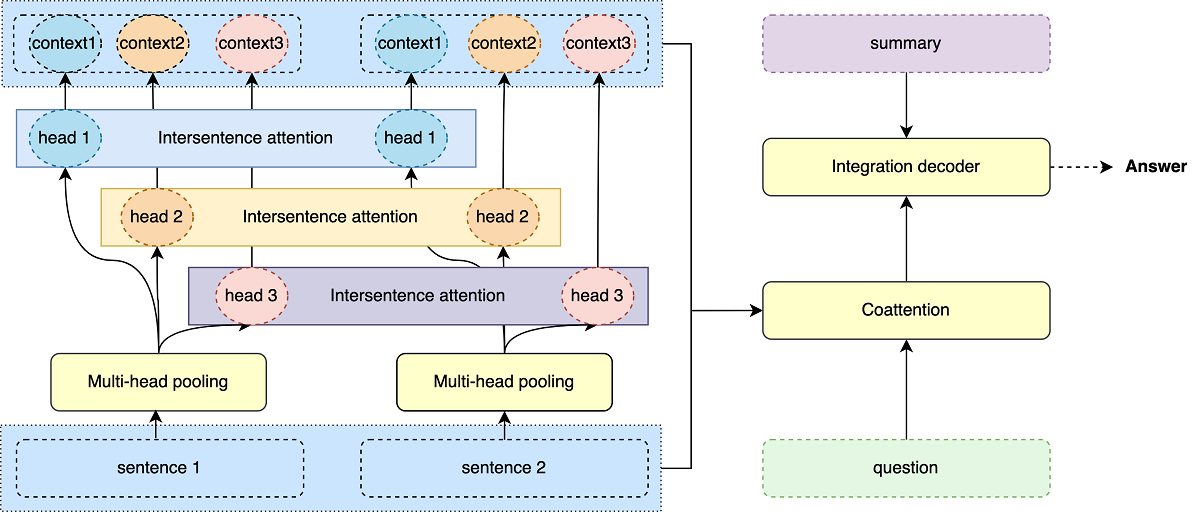
Overview of our model.

For the text summarization task, formally, assume that we have a question *q* = {*q*_1_, ..., *q_m_*} with m words and a source document 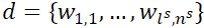 containing *l^s^* sentences that have *n^s^* words at most. The task is to generate an answer summary *y* = {*y*_1_, ..., *y_n_*} containing *n* words. The training goal is to maximize the probability *p*(*y*|*q*, *d*). The overall architecture of our transformer-based question-driven abstractive answer summarization model is depicted in Figure 1, which consists of three main components: (1) two-step attention mechanism, (2) overall integration mechanism, and (3) multi-view pointer network for generation.

For the QA task, given a question *q* and an answer summary *y*, the model should generate an answer *a* = {0,1} indicting yes or no to this question conditioned on the document. We adopted BioBERT [23] as our model to evaluate the factual consistency, which is initialized with bidirectional encoder representation from transformers (BERT) [24] and further pretrained on large-scale biomedical corpora.

### Encoder

#### Question Encoder

Let 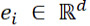 denote the token embedding indicating the meaning of each token *q_i_*. A special positional encoding *pe_i_* indicates the position of each token within the question sequence. The input of the question encoder *I^q^* is a sequence of embeddings.

A transformer layer is used to encode the question. It reads the question *q* = {*q*_1_, ..., *q_m_*} and computes a hidden representation 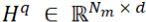, where *N_m_* denotes the length of the question and *d* is the dimension of the vector. To get a fixed length question representation, *H^q^* is then converted to a vector 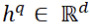 by adding all token representations and normalizing it by question length.



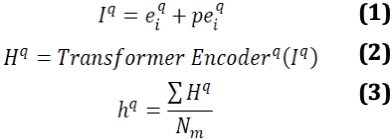



#### Sentence Encoder

Each document is composed of several sentences. Given a document context 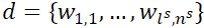, the input of the sentence encoder is the sentences fed one by one. We used sentence position embedding to indicate the order of sentences.



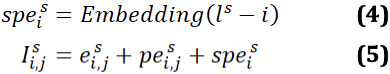



where 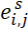 is the word embedding of *w_i,j_*, which is the same word embedding as 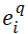; the position embedding of the token is represented as 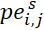, and 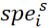 denotes the sentence position embedding of 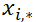.

*I^s^* then fed into a transformer encoder to represent the sentence as a sequence of hidden vectors by:



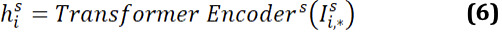



The hidden representation of a document is represented as 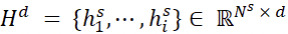 and a sentence vector 
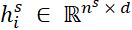
, where *N^s^* = *l^s^* × *n^s^*.

### Two-step Attention Mechanism

#### Intersentence Attention

Inspired by Liu and Lapata [25], we used an intersentence attention mechanism to model the dependencies across multiple sentences, where each sentence can attend to other sentences. We used a weighted-pooling operation to obtain a fixed-length sentence representation so that the diversity of each sentence representation is increased. Through a *multi-head pooling mechanism* [25], each token can attend to other tokens by calculating weight distributions. Sentences can be encoded flexibly in different subspaces.

The output representation 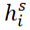 of the last transformer encoder layer for token *w_i_*_,_*_j_* is denoted as *x_i_*_,_*_j_* as the input. For each sentence 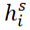 and for head *z* ∈ {1, ..., *n_head_*}, we first conducted a linear transformation to obtain the attention scores 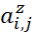 and value vectors 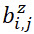. The probability distribution 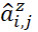 was then calculated within the sentence.



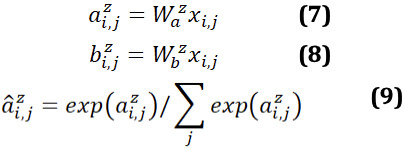



where 
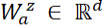
 and 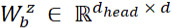 are weights. *d_head_* = *d* / *n_head_* is the dimension of each head.

Based on the probability distributions and value vectors, we conducted a weighted summation followed by another linear formation and layer normalization. Different vector 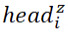 encodes sentences in a different subspace.



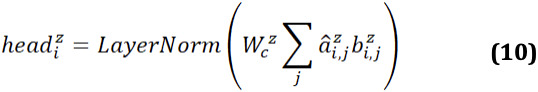



where 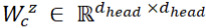 is the weight. Because of the flexibility of combining multiple heads, each sentence has multiple attention distribution and focuses on different views of input.

Dependencies among multiple sentences can be modeled by the intersentence attention that is similar to self-attention. Intersentence attention computes the distribution of attention so that each sentence attends to other sentences.



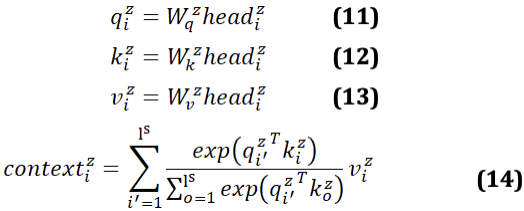



where 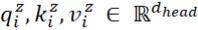 are query, key, and value vectors, respectively. Through a self-attention calculation, 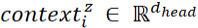 is obtained to represent the sentence vector that gathers the information of other sentences. l^s^ is the number of input sentences.

We then concatenate all context vectors and pass through a linear layer with weight 
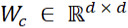
 to update token representations by adding *c_i_* to each token vector *x_i_*_,_*_j_*. We then pass it through a two-layer multilayer perception, taking *gelu* as the activation function [26]. Next, we pass the summation of *x_i_*_,_*_j_* and *g_i_*_,_*_j_* to a layer normalization. In this way, each sentence collects information from other sentences represented as 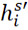.



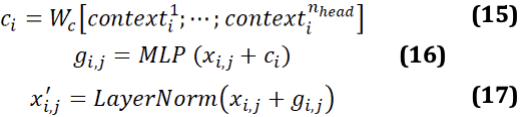



#### Coattention

Coattention is the second attention mechanism aimed at exploiting the pairwise mutual information between the question and the context.

We further used an additive attention [27] to obtain the distribution of document sentences that highly coincides with the question and then combines the question and question-related sentences to get their comprehensive representation 
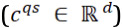
 by:



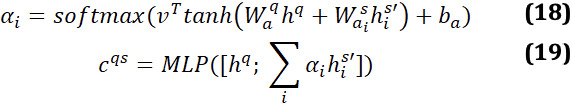



where *MLP* is the same as mentioned before. 
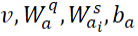
 are trainable parameters.

### Integration Decoder

When given the first *t* – 1 tokens in the summary *y*_1_, ..., *y_n_*, the integration decoder incorporates the question and the document into the summary through an overall integration mechanism. The purpose is to predict the representation of the *t* – *th* token and transmit it to the pointer network.

#### Overall Integration

Inspired by gated recurrent units [28], we designed an *integration* gate (*z*) to integrate the question-document and summary, which enables summary tokens at different times to merge information in different levels. Multi-head attention is then used to capture the information in the fused representation, 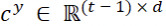, and obtain *s^y^*, which is a correlative summary. 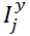 is the vector representation of the input summary.



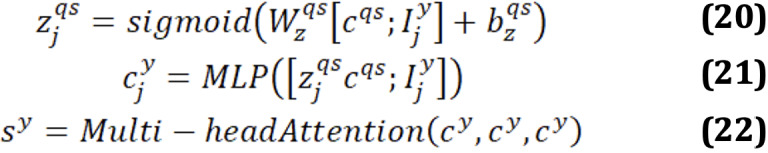



To reinforce the understanding of the question and document of the decoder, *s^y^* is used to compute attention with the question and the document, and obtain representations *s^q^* and *s^s^*.

*s^q^* = *Multi* – *headAttention* (*s^y^*, *H^q^*, *H^q^*) **(23)**

*s^s^* = *Multi* – *headAttention* (*s^y^*, *H^s^'*, *H^s^'*) **(24)**

Next, similar to equation 20, the predicted representation *o^y^* is obtained to integrate the attentive question, the attention document, and the correlative summary by using the *integration* gate.



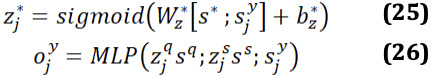



where * is denoted as *^q^* or *^s^*.

### Multi-View Pointer Network

**Figure 2:**
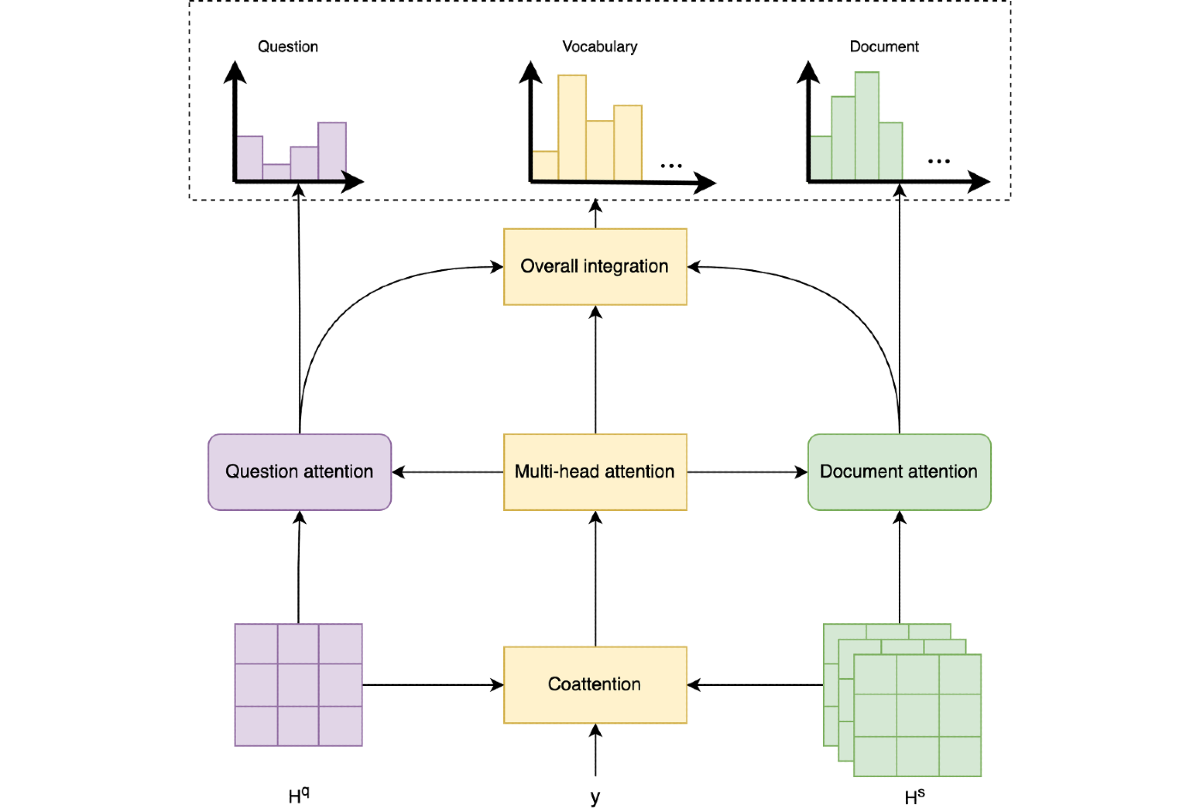
Multi-view pointer network. H^q^: hidden representation of question; y: hidden representation of the input summary; H^s^: hidden representation of document.

To improve the probability of generating corresponding tokens from the question and the document, a novel multi-view pointer network is proposed based on multi-head attention as shown in Figure 2.

#### Question Tokens

We computed the attention weights *β^q^* through multiple attention weights in the multi-head attention.



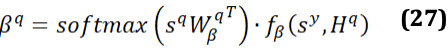



Where *f_β_* means a function of getting multiple attentions in the multi-head attention. 
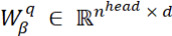
 is the weight, where *n^head^* is the number of heads. *β^q^* can be treated as the probability distribution over the question words. It can be represented as 
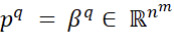
.

#### Document Tokens

The distribution of the document that is relevant to the question can be served as a global distribution over each decoding step. *β^s^* can be calculated similar to equation 27, which can be considered a local distribution at each decoding step. Thus, the distribution over the document can be calculated by:



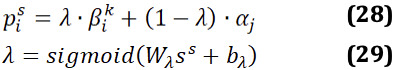





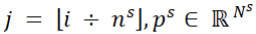



#### Vocabulary Tokens

The predicted representation from the overall integration decoder is used to calculate the probability distribution *p^v^* over the fixed vocabulary through a *softmax* layer; *W^v^* is the weight from the word embeddings.



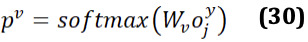



The final probability distribution *y_t_* to predict can be formulated from three aspects of word distributions as:

*P*(*y_t_* |*q*, *d*, *y* < *t*) = *softmax* (*W_γ_o^y^* + *b_γ_*) ⋅ [*p^v^*, *p^q^*, *p^s^*] **(31)**

#### Loss Function

The main training objective is to minimize the negative log likelihood between the reference summary and the predicted summary. Thus, Trans-Att can be trained by minimizing the objective.



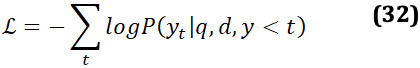



### Question-Answering Model

BERT [24] has already been used in QA tasks. We fine-tuned BioBERT [23] as a baseline. We fed PubMedQA questions and corresponding texts that could be contexts, reference long answers, contexts and long answers, or generated summaries for comparison, separated by special [SEP] token, to the model. We take the special embedding [cls] from the last layer and use a *softmax* function to predict the final label that could be yes or no. The general loss was trained by minimizing the cross-entropy between the predicted labels and the true label distribution.

## Results

### Data Set

We evaluated our model on the nonfactoid QA data set PubMedQA [6]. PubMedQA is a novel biomedical data set aiming at answering academic questions and has substantial instances with some expert annotations. Each instance is composed of a question that is a general question, a context that is the structured abstract without its conclusion, a long answer that is the conclusion of the abstract in terms of the question, and a final answer yes/no for the general question that summarizes the conclusion and can be used for the QA task. The statistics of the PubMedQA data set are shown in Table 1.

We adopted ROUGE-1, ROUGE-2, and ROUGE-L to automatically evaluate the summarized answers in the question-driven abstractive summarization task. The main metrics of the QA task are accuracy and macro-F1 under a reasoning-free setting in which the generated summary is added in the input.

**Table 1:** Statistics of the PubMedQA data set.

Task data set	Training, n	Development, n	Test, n
QA^a^ pairs	169,000	21,000	21,000
Average question length (word count)	16.3	16.4	16.3
Average document length (word count)	238	238	239
Average summary length (word count)	41.0	41.0	40.9
Average number of sentences	9.32	9.31	9.33

^a^QA: question-answering.

### Experimental Settings

ParlAI [29] was implemented in our model as the code framework. The dimensions of word embedding size and hidden size were both 256. The text was encoded by byte-pair encoding [30], and the embedding matrix was initialized with fastText. Both encoder and decoder layers of transformer-based models were 5, with feed-forward hidden size 512 and attention head 4 for all layers. The optimizer was Adam [31] with an initial learning rate of 0.0005. We also applied the inverse square root learning schedule over the 5k warm-up dates. The dropout rate was set to 0.2, and gradient clipping was used with a maximum gradient norm of 0.1. Label smoothing of the value 0.1 was used for summary generation. We used beam search in the generation process with beam size 2 and adopted 3-gram blocking.

### Comparative Methods

We report the performance of our proposed model in comparison with several baselines and state-of-the-art methods based on different methodologies, including extractive summarization, abstractive summarization, query-based summarization, and question-driven abstractive summarization.

Two unsupervised extractive methods were used. LEAD3 is a simple but effective extractive summarization baseline that concatenates the first two sentences and the last sentence without question information. Maximal marginal relevance is an information retrieval model used to calculate the similarity between the text and the researched document for extractive summarization.

Three widely adopted abstractive methods were adopted for comparison. Sequence-to-sequence model with attention [27] is a simple encoder-decode model with attention based on RNN without respect to the question. Pointer-generator network [21] is a hybrid pointer-generator architecture with coverage based on a neural sequence-to-sequence model for abstractive text summarization. Transformer [15] implements the state-of-the-art encoder-decoder framework based on multi-head attention without access to the question.

There were two query-based abstractive summarization methods used for comparison. The soft long short-term memory–based diversity attention model (SD_2_) [10] adds a query attention mechanism to a sequence-to-sequence model. It learns to pay attention to different parts of the query at different time steps. Query-based summarization using neural networks (QS) [11] incorporates question information into the pointer-generator network with the use of the vanilla attention mechanism.

Finally, we implemented two of the latest question-driven answer summarization models for comparison. Hierarchical and sequential context modeling [7] is a hierarchical compare-aggregate method used to integrate the interaction between the question and the document into final document representation at both the word level and sentence level. Multi-hop selective generator (MSG) [8] models the relevance between question and sentences by leveraging a humanlike multi-hop reasoning process for question-driven summarization, in which the most related sentences are given higher weights.

### Experimental Results

The experimental results of question-driven summarization in terms of ROUGE scores and QA with respect to accuracy and macro-F1 scores are presented in Tables 2 and 3. Both ROUGE scores and metrics of QA show that our model achieved competitive performance in comparison with state-of-the-art question-driven summarization methods.

Compared with traditional text summarization, there was limited improvement for query-based summarization methods (SD_2_ and QS), indicating that the question information was not sufficiently used. There was a noticeable margin, about 0.79 for ROUGE-2, higher than the current state-of-the-art model (MSG). This indicates that the model benefits from the information provided by mutual information between question and document, and among sentences. We noticed that the ROUGE-1 score of our model was lower than MSG. One possible explanation is that the length of the generated summary of MSG was longer than that of our model. Considering the characteristic of ROUGE-1 that measures the word overlap between the reference summary and the predicted summary, the longer summary has more possibility of generating words that appeared before.

As for the QA result, we observed that if using the original answer summary, BioBERT achieves good enough scores. If the input answer summary can correctly answer the question, it is consistent to the original semantics. Thus, evaluating the factual consistency by a QA task is feasible. Suppose that we feed the context without long answer information to the model, which is under the reasoning-required setting; the result is comparatively lower because the reasoning and inference process is crucial in answering the question if the answer is not directly available. We treated the long answer as the summary, and its quality influenced the factual consistency. It was observed that there is still a big gap between the generated summary and the reference summary, which leaves room for improvement.

Overall, the difference upon accuracy measurement was not significant by a narrow margin because of the imbalanced distribution of labels (92.8% yes vs 7.2% no). The F1 score was significant and representative, and our model achieved the best *F* score of 77.57%. The results show that the extractive methods performed better than the abstractive methods. We speculate that extractive summarization approaches directly copy from the source context. However, it is worth noting that the extractive methods have an upper bound, and they barely exceed the performance when given the whole context. There is substantial potential for abstractive approaches. Future work should explore the reasoning ability of abstractive methods.

**Table 2:** Comparison with related works of question-driven summarization task.

Methods	Types	With question	ROUGE^a^-1 (%)	ROUGE-2 (%)	ROUGE-L (%)
LEAD3	Extractive	No	30.94	9.79	25.89
MMR^b^	Extractive	No	29.69	9.50	24.10
S2SA^c^	Abstractive	No	32.40	11.00	27.30
PGN^d^	Abstractive	No	32.89	11.51	28.10
Transformer	Abstractive	No	32.38	11.34	26.32
SD_2_^e^	Abstractive	Query based	32.33	10.52	26.01
QS^f^	Abstractive	Query based	32.60	11.10	26.70
HSCM^g^	Extractive	Question driven	32.34	10.07	25.98
MSG^h^	Abstractive	Question driven	*37.20* ^i^	14.80	30.20
Trans-Att (ours)	Abstractive	Question driven	36.01	*15.59*	*30.22*

^a^ROUGE: recall-oriented understudy for gisting evaluation.

^b^MMR: maximal marginal relevance.

^c^S2SA: sequence-to-sequence model with attention.

^d^PGN: pointer-generator network.

^e^SD_2_: soft long short-term memory–based diversity attention model.

^f^QS: query-based summarization using neural networks.

^g^HSCM: hierarchical and sequential context modeling.

^h^MSG: multi-hop selective generator.

^i^Italics indicate the best result.

**Table 3:** Comparison with related work for question-answering task.

Methods	Accuracy (%)	F1 (%)
LEAD3	93.80	67.06
MMR^a^	*94.85* ^b^	75.69
S2SA^c^	91.89	63.81
PGN^d^	91.93	64.42
Transformer	94.18	69.59
SD_2_^e^	94.34	69.30
HSCM^f^	93.78	76.48
MSG^g^	93.68	73.27
Trans-Att (ours)	94.20	*77.57*
Majority	92.76	48.12
Context	96.50	84.65
Long answer	99.04	96.18
Context + long answer	99.20	96.86

^a^MMR: maximal marginal relevance.

^b^Italics indicate the best result.

^c^S2SA: sequence-to-sequence model with attention.

^d^PGN: pointer-generator network.

^e^SD_2_: soft long short-term memory–based diversity attention model.

^f^HSCM: hierarchical and sequential context modeling.

^g^MSG: multi-hop selective generator.

### Ablation Study

To examine the contributions of our proposed modules, namely, intersentence attention, coattention, overall integration, and multi-view pointer network, we ran an ablation study. The experimental results are shown in Table 4.

Overall, all the modules contributed to the final performance to some extent. The accuracy score was not significant compared with the F1 score because of the imbalanced distribution of labels. When the coattention was discarded, the performance of the model dropped substantially, which indicates that it plays a more important role in exploiting the pairwise mutual information between the question and the document sentences. Besides, applying intersentence attention also improved the performance, which indicates that it is not enough to only consider the question-related information. Interrelation among sentences is also worth paying attention to. The decrease on F1 was most significant, which demonstrates the effects of the two-step attention mechanism. Overall integration reinforces the understanding of the model upon the question and the document indicated by a noticeable decrease in F1. Because of the biomedical characteristic of PubMedQA, the out-of-vocabulary problem is much more severe. The ablation study results validated the importance of the multi-view pointer network.

**Table 4:** An ablation study for our model.

Methods	ROUGE^a^-1	ROUGE-2	ROUGE-L	Accuracy (%)	F1 (%)
Trans-Att	36.01	15.59	30.22	94.20	77.57
Intersentence attention	34.65	13.92	28.07	93.87	73.13
Coattention	34.05	13.61	26.50	93.40	70.62
Overall integration	34.28	14.26	28.63	94.53	72.37
Multi-view pointer network	35.16	13.98	29.32	94.39	75.67

^a^ROUGE: recall-oriented understudy for gisting evaluation.

### Case Study

**Figure 3:**
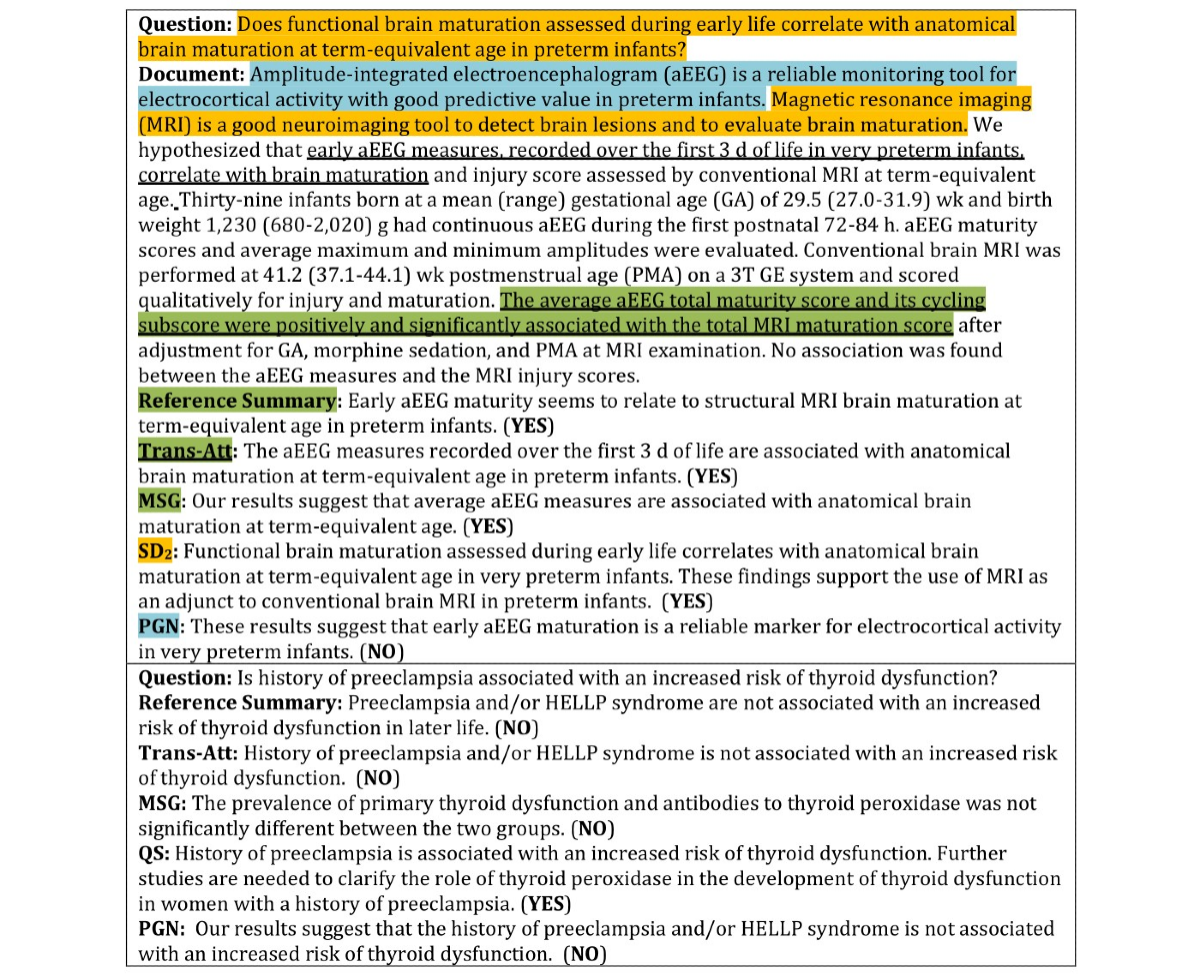
Case study from PubMedQA (the bottom example omits the context; final answer is in parentheses). MSG: multi-hop selective generator; PGN: pointer-generator network; QS: query-based summarization using neural networks; SD_2_: soft long short-term memory–based diversity attention model; HELLP: hemolysis, elevated liver enzymes, and low platelets counts syndrome.

In Figure 3, we show the summaries generated by the proposed method and some baseline methods for comparison, and visualize the sources of the summaries with colors. The context underlined and highlighted with green was used by Trans-Att to generate the summary, which contains more information than in the reference summary. By comparison, we observed that Trans-Att not only successfully exploits the intersentence information with useful information but also uses the question information in understanding semantic content; pointer-generator network generates an irrelevant summary, which proves the importance of the question information; SD_2_ fails to capture the core argument, resulting in repeating the question and paying attention to wrong information; the final answer demonstrates the validity in evaluating factual consistency by QA task (although SD_2_ gives the right final answer, there is still a semantic mismatch because the first sentence is essentially the same as the question); and the bottom example demonstrates that there are limitations to the yes/no questions, the answer of which depends partly on clues of negative pronouns. Future work will consider increasing the diversity of the QA task.

### Novel N-Grams

We also analyzed the output of abstractive models by calculating the proportion of novel n-grams that appear in the summaries but not in the source texts. Table 5 shows that summaries of our model account for a lower rate of novel n-grams than the reference summaries, indicating the quality of abstraction. We observed that the traditional abstractive approach (pointer-generator network), copies more phrases, perhaps because it generates more words from the context without being question driven, which increases the probability of unrelated information being selected. Note that MSG produces novel n-grams more frequently. However, it may contain the factual inconsistency problem in generating new words.

**Table 5:** Proportion of novel n-grams.

Methods	1 grams (%)	2 grams (%)	3 grams (%)	4 grams (%)
Trans-Att	11.00	47.82	67.12	79.38
MSG^a^	13.43	54.66	74.13	85.01
PGN^b^	16.29	43.73	58.38	69.14
Refrence	27.83	72.11	87.17	93.55

^a^MSG: multi-hop selective generator.

^b^PGN: pointer-generator network.

## Discussion

### Conclusions

In this paper, a novel transformer-based question-driven abstractive summarization model was proposed to generate concise and consistent summaries for nonfactoid QA. A two-step attention mechanism was proposed to exploit the mutual information both of the question to context and the sentence over other sentences. We used the overall integration mechanism and the novel pointer network to better integrate and use information of the question, document, and summary. We conducted a QA task to evaluate the factual consistency between the generated summary and the reference summary. Experimental results demonstrate that our proposed model achieves comparable performance to the state-of-the-art methods.

### Future Work

Due to the insufficiency of the data set quantity, we were limited to conducting experiments on PubMedQA. We are looking forward to conducting more persuasive experiments when the insufficiency is lifted. As for the evaluation of the factual consistency, we can also incorporate human expertise to further enhance the credibility of the proposed QA metric. Hopefully, our method can provide some inspiration in the summarization task.

## Abbreviations

BERT: bidirectional encoder representation from transformers
MSG: multi-hop selective generator
QA: question answering
QS: query-based summarization using neural networks
RNN: recurrent neural network
ROUGE: recall-oriented understudy for gisting evaluation
SD_2_: soft long short-term memory–based diversity attention model
